# Exploring the Psychosocial Effects of Male Infertility and the Experiences of Healthcare Workers in Fertility Centers in Osogbo, Nigeria: A Qualitative Study

**DOI:** 10.7759/cureus.96221

**Published:** 2025-11-06

**Authors:** Kehinde Awodele, Sunday C Adeyemo, Adeoye Oyewole, Adeniyi Fasanu, Eniola D Olabode, Ayodele R Ajayi, Sunday Olarewaju, Babalola O Emmanuel, Samuel O Omopariola, Ayodeji O Oyeniran

**Affiliations:** 1 Obstetrics and Gynaecology, Osun State University, Osogbo, NGA; 2 Public Health Research, University of Wolverhampton, Wolverhampton, GBR; 3 Health and Biomedical Sciences, Institut Superieur de Santé, Niamey, NER; 4 Psychiatry Medicine, Ladoke Akintola University of Technology, Ogbomosho, NGA; 5 Community Medicine, Institut Superieur de Santé, Niamey, NER; 6 General Nursing, Pear Tree Court Care Home, Care UK, Waterlooville, GBR; 7 Psychiatry, Afe Babalola University Teaching Hospital, Ado-Ekiti, NGA; 8 Community Medicine, Osun State University, Osogbo, NGA; 9 Obstetrics and Gynaecology, Obafemi Awolowo University, Ile-Ife, NGA; 10 Obstetrics and Gynaecology, All Women's Care Fertility and Specialist Hospital, Osogbo, NGA; 11 Obstetrics and Gynaecology, Obafemi Awolowo Teaching Hospital Complex, Ile-Ife, NGA

**Keywords:** emotional, infertility, male, osogbo, psychosocial

## Abstract

Background: Infertility is a major public health concern worldwide. There remain gaps in qualitative research specifically exploring the psychosocial experiences of men dealing with infertility. This study, therefore, explored the psychosocial and emotional effects of male infertility and examined the experiences and challenges faced by healthcare workers involved in the management of male infertility centers in Osogbo, Nigeria.

Method: The study was a qualitative study carried out among male patients attending fertility centers and health workers in Osogbo. Inclusion criteria include male patients who have been engaged in deliberate attempts to achieve pregnancy and are seeking medical assistance in the selected fertility centers, as well as health workers currently involved in infertility management for at least six months. Exclusion criteria include men and health workers who are ill or unwilling to participate. The study employed a two-stage purposive sampling approach. Data were collected through Focus Group Discussions (FGDs) among male patients and In-Depth Interviews (IDIs) with healthcare workers. Informed written consent was obtained from participants. Data from the discussions and interviews were recorded, transcribed, and analyzed using the ATLAS.ti software (Lumivero, LLC, Denver, CO, USA). Thematic analysis was employed to categorize responses into different themes.

Result: All the respondents acknowledged that male infertility can lead to psychological, emotional, and social distress for the family. Health workers encountered challenges in giving health talks, including denial and unwillingness to accept the results, feeling embarrassed, and the belief that the women should be the cause of infertility and not the male. Similarly, it is extra difficult to counsel men who have fathered a child before and are infertile.

Conclusion: The findings highlight the necessity for healthcare systems to adopt comprehensive strategies that address both the medical and psychosocial aspects of male infertility.

## Introduction

Infertility is a major public health concern worldwide, affecting an estimated 10-15% of couples trying to conceive. It is defined as the inability of a sexually active couple to achieve pregnancy within one year without using contraceptives [1]. Although male-factor infertility contributes to approximately 40-50% of cases, it is far less studied in many low- and middle-income settings compared to female infertility [2]. Though infertility has been historically perceived as a woman's problem, recent research shows that male infertility is also a major cause and needs more attention. A recent study among male patients in Osogbo shows that more than 80% of the respondents had at least one abnormality in their seminal fluid [3].

In African contexts, the effect of infertility is worsened by cultural norms and gender expectations, which lead to social stigma. A scoping review of psychosocial infertility studies in Africa showed that recurring themes include stigma, withdrawal from social functions, and reduction in spousal and family relationships [2,4].

Male infertility leads to substantial psychosocial and emotional burdens. Men often experience depression, anxiety, lowered self‐esteem, feelings of inadequacy, shame, and social isolation when diagnosed with infertility. For example, in a qualitative study of men undergoing intracytoplasmic sperm injection (ICSI), participants described emotional distress, strained partner relationships, and selective disclosure of their condition [5].

Healthcare providers are also affected indirectly; they face challenges in counseling male patients about infertility, addressing psychosocial distress, and bridging gaps in patient education. In Nigeria, studies have documented a lack of awareness, misconceptions, and psychological distress among infertile couples, which places demands on clinicians for clearer communication and more empathetic care [6].

Though studies have explored the medical and biological aspects of male infertility, there remain gaps in qualitative research specifically exploring the psychosocial experiences of men with infertility from both patient and provider perspectives in fertility center settings in Nigeria. A deeper understanding of these experiences will inform more culturally sensitive interventions, improve counseling practices, and enhance the well-being of affected men.

The specific objectives of the study include (1) to explore the psychosocial and emotional effects of male infertility and (2) to examine the experiences and challenges faced by healthcare workers in fertility centers managing male infertility in Osogbo, Nigeria.

Findings from the study are intended to inform psychosocial interventions, counseling strategies, and healthcare practices within fertility centers. By identifying key emotional challenges and professional barriers, the research aims to provide evidence that can improve patient support, enhance counseling effectiveness, and guide policy development in reproductive health services.

## Materials and methods

The study was a qualitative study carried out among male patients attending fertility centers and health workers in Osogbo. Inclusion criteria included male patients within the age range of 18 to 50 years, male individuals seeking medical assistance for fertility-related issues in the selected study centers, and who have been engaging in deliberate attempts to achieve pregnancy with their partners for a minimum of one year without achieving success. Inclusion criteria for healthcare workers included doctors or nurses currently involved in infertility management with at least six months of work experience in infertility care, and who are willing to participate and provide informed consent. Exclusion criteria included infertile men who declined participation or were too ill to attend discussions, and health workers who were not directly involved in infertility management.

The study employed a two-stage purposive sampling approach. In the first stage, five out of 10 fertility centers in Osogbo were purposively selected based on their patient volume, accessibility, and involvement in infertility management. The centers included were UNIOSUN Teaching Hospital (UTH), Hallelujah Specialist Hospital (HSH), Ayomide Fertility Centre (AFC), SW Aqua Fertility Centre (SWA), and Crystal Medical and Specialist Centre (CMS). These facilities were chosen to ensure a broad representation of both public and private fertility services in the city. In the second stage, participants were purposively selected from the identified centers. Purposive sampling ensured that only participants with direct experience and relevant knowledge of male infertility were included. Specifically, men who had been clinically diagnosed with infertility were considered to have direct experience, while healthcare workers such as fertility specialists, nurses, and counselors with at least one year of experience providing care for infertile men were deemed to have relevant knowledge.

Data were collected from June 20 to 30, 2024, through Focus Group Discussions (FGDs) among male patients and In-Depth Interviews (IDIs) with healthcare workers. One FGD was conducted in each fertility center, comprising six participants per group. A total of 10 healthcare workers (two from each fertility center) were interviewed individually to obtain their professional perspectives. The number of FGDs and interviews conducted was guided by the principle of data saturation, that is, when no new themes emerged from the discussions. Participants were recruited with the assistance of clinic managers and fertility nurses who identified eligible individuals. All participants were informed about the purpose of the study, assured of confidentiality, and provided written informed consent prior to participation. The number of sessions was guided by data saturation, and each lasted approximately 60-90 minutes.

Discussion guides were developed in line with the study objectives and covered key themes, such as emotional and psychosocial effects of infertility, coping mechanisms, healthcare experiences, and recommendations for improving patient support and counseling. All sessions were audio-recorded, transcribed verbatim, and analyzed using ATLAS.ti software (Lumivero, LLC, Denver, CO, USA). Thematic analysis followed Braun and Clarke's six-step framework, involving familiarization with the data, generation of initial codes, theme identification, and refinement through iterative discussion between two independent researchers. Credibility and trustworthiness were enhanced through peer debriefing, researcher triangulation, and member checking with selected participants to validate emerging themes and interpretations.

Ethical approval for this study was obtained from the Ethics and Research Committee of Osun State University Teaching Hospital (UTH/EC/2024/06/957). Approvals were also obtained from the management of each facility. All ethical principles guiding the conduct of research, such as informed consent, beneficence, non-maleficence, confidentiality, justice, and autonomy, were strictly taken into consideration.

## Results

Table 1 presents the sociodemographic characteristics of the study participants, consisting of 30 infertile men and 10 healthcare workers. Among the infertile men, the largest proportion (12, 40.0%) were aged 30-39 years. In terms of education, 20 (66.7%) of the infertile men had attained tertiary education. In terms of occupation, for infertile men, 15 (50.0%) were businessmen or traders. Among healthcare workers, there were 5 (50.0%) doctors, 3 (30.0%) nurses, and 2 (20.0%) laboratory technicians. Regarding the years of experience of the healthcare workers, 4 (40.0%) had 6-10 years of experience out of the 10 healthcare workers (Table 1).

**Table 1:** Sociodemographic characteristics of the study participants Data have been presented as frequencies and percentages. HCW: Healthcare worker

Sociodemographic Characteristic	Category	Infertile Men (n = 30), n (%)	Healthcare Workers (n = 10), n (%)
Age (years)	20–29	6 (20.0)	1 (10.0)
	30–39	12 (40.0)	5 (50.0)
	40–49	10 (33.3)	4 (40.0)
	≥50	2 (6.7)	0 (0.0)
Education level	Primary	0 (0.0)	0 (0.0)
	Secondary	10 (33.3)	0 (0.0)
	Tertiary	20 (66.7)	10 (100.0)
Occupation/role	Unemployed/Other	5 (16.7)	-
	Skilled/Professional	10 (33.3)	-
	Business/Trader	15 (50.0)	-
	Doctor	-	5 (50.0)
	Nurse	-	3 (30.0)
	Laboratory Technician	-	2 (20.0)
Years of experience (HCWs only)	0–5	-	3 (30.0)
	6–10	-	4 (40.0)
	>10	-	3 (30.0)

Emerging themes

**Figure 1:**
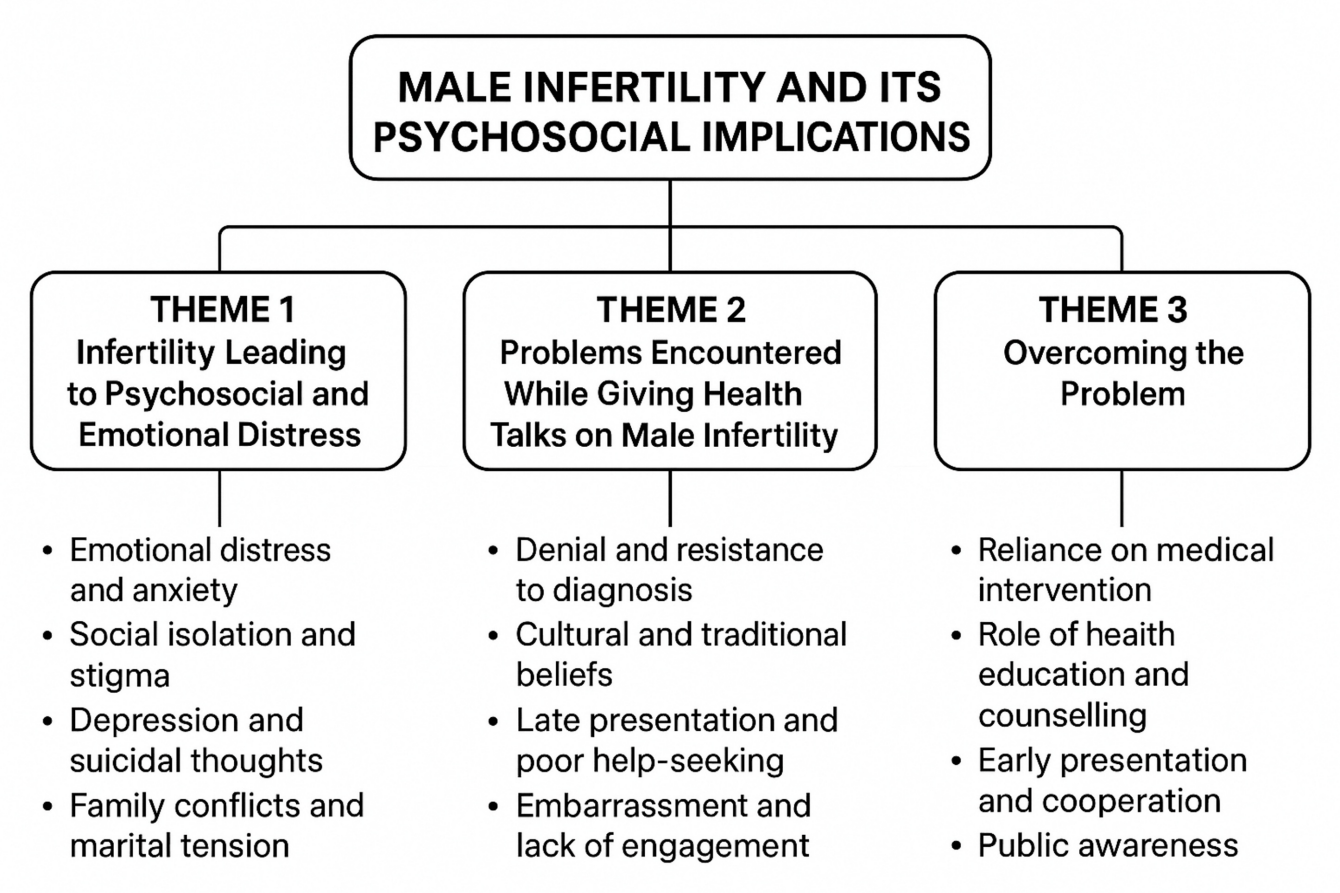
Themes and sub-themes from the focused group discussions and interviews

There are three major emerging themes with sub-themes from the focused group discussions and interviews (Figure 1).

Infertility Leading to Psychosocial and Emotional Distress

All the respondents acknowledged that male infertility can lead to psychological, emotional, and social distress to the family, as the male will not want a divorce from his wife, and they may indulge in excessive thinking and start withdrawing from people that they used to or should be moving with.

Problem Encountered While Giving Health Talk to Men With Infertility

According to the health workers, which include nurses and doctors, problems encountered in giving health talks include denial, unwillingness to accept the results, feeling embarrassed, and the belief that women should be the cause of infertility and not men. It is extra difficult to counsel a man who has fathered a child before and is infertile.

Overcoming the Problem

The non-health workers among the participants believed that doctors can find a solution to infertility problems if patients undergo regular medical checkups. In contrast, doctors and nurses primarily believed in health education for patients and treatment as a means of overcoming the problems of male infertility (Table 2).

**Table 2:** Participant quotes by themes HSH: Hallelujah Specialist Hospital; UTH: UNIOSUN Teaching Hospital; SWA: SW Aqua Fertility Centre

Theme/Subtheme	Participant Quotes
Infertility Leading to Psychosocial and Emotional Distress	“Yes, it can … it can lead to emotional and social distress to the family…" (Male Patient, HSH, 30–39 years)
	“They may initially think it was due to the woman factor. If the man is now diagnosed as infertile, he is automatically the cause. Socially, they will not want their wife to divorce them, and some may even … so many problems, emotionally. Some men may even commit suicide.” (Male Patient, UTH, 40–49 years)
	“Yes, emotional problems, they will be thinking about it. For example, it causes social distress as you won’t be able to move with people that you are supposed to move with.” (Male Patient, SWA, 20–29 years)
	“It causes lots of thinking and affects the family, especially those that have had children before. It can even lead to controversies and family issues.” (Male Patient, CMS, 30–39 years)
Problem Encountered While Giving Health Talk on Male Infertility	“Most of the patients would not want to accept that they are being infertile. And that's the main problem…the acceptance.” (Doctor, Female, 30–39 years)
	“There is this problem of denial; most of them want to deny the fact that they are infertile, even with the test results showing low sperm counts they still tend to deny it.” (Laboratory Technician, Male, 20–29 years)
	“The problems, you know, to sit a man down and tell him he is the cause. You want to educate him on what to do. And most of these infertile males, by the time it is discovered, they are late …” (Doctor, Female, 30–39 years)
	“They may not have time for you or they think it's embarrassing educating them.” (Nurse, Female, 30–39 years)
	Well, sometimes the traditional or cultural beliefs, for example, some people don't believe that a couple with infertility; they always believe it's the woman's fault … Maybe they've also fathered a child in the past, whereas changes could have occurred afterwards. They find it difficult to accept infertility.” (Doctor, Male, 30–39 years)
Overcoming the Problem	“The doctor knows what to do, the right thing. And I believe by the special grace of God and the power of the doctors, I think we should see solutions to it … my own advice is that we should go for medical check-up.” (Male Patient, UTH, 40–49 years)
	“I think by reassuring them that there is a solution … if they know that there is a solution, they can come to acceptance and work together to get a solution for the infertility. By proper health education, letting them know that it can be managed if they present early enough depending on the underlying cause, letting them know that there are various causes of infertility …” (Doctor, Female, 30–39 years)
	“For males, I think through medical investigations and any conditions that are investigated and found should be treated accordingly. The best way to tackle the obstacle is public enlightenment against harmful lifestyles and practices such as smoking and alcohol consumption …” (Doctor, Male, 30–39 years; Nurse, Female, 30–39 years)

## Discussion

This study explored the psychosocial impact of male infertility in Osogbo, Nigeria, highlighting significant emotional and social distress among infertile men, as well as challenges faced by healthcare workers in providing effective counseling.

The findings indicate that male infertility leads to considerable psychological distress, including emotional turmoil and social withdrawal. Participants reported feelings of shame and excessive rumination, with some even contemplating suicide. These findings are consistent with recent meta-ethnographic research showing that men across diverse cultural contexts experience emotional trauma, a sense of failure, and reduced self-esteem as a result of infertility [7].

Male infertility was also reported to impact marital stability and family cohesion. Participants emphasized men's reluctance to acknowledge their role in childlessness, often leading to denial, family tension, and fear of divorce. The stigma associated with male infertility in Nigeria often results in the condition being perceived as a female issue, leading to delayed diagnosis and treatment. This societal bias contributes to the emotional burden on men, as they may feel isolated and unsupported [8].

Healthcare providers reported encountering denial and embarrassment among male patients when discussing infertility. Many patients were reluctant to accept their diagnosis, especially if they had previously fathered children. This resistance complicates efforts to provide effective counseling and education on infertility management [9]. These challenges are consistent with findings from other Nigerian studies, where healthcare workers noted difficulties in addressing male infertility due to cultural beliefs and patient denial [10]. The reluctance to acknowledge male infertility underscores the need for targeted interventions to educate both patients and healthcare providers.

Participants suggested that regular medical checkups and professional counseling could alleviate the distress associated with infertility. Healthcare workers emphasized the importance of health education, early diagnosis, and appropriate treatment to manage male infertility effectively.

Recent literature supports these recommendations, advocating for a holistic approach that integrates psychological support into infertility care. Interventions such as cognitive-behavioral therapy, mindfulness techniques, and fertility counseling have been shown to reduce stress and improve psychological resilience among patients undergoing fertility treatments [7].

Strengths and limitations of the study

The qualitative design of the study and its integration of both patients and healthcare workers allowed for rich, in-depth exploration. However, the use of a small, purposive sample, though appropriate for in-depth exploration, may limit representativeness and generalizability and might have introduced selection bias. The findings of this study are context-specific and may have been influenced by social desirability as well as cultural norms and societal expectations surrounding male infertility in Osogbo, Nigeria. Participants' willingness to share experiences and the interpretation of their responses by the researchers may reflect these local cultural dynamics, which may limit the transferability of the results to other settings.

Recommendations

Fertility clinics should provide culturally sensitive psychological support for men, while health authorities implement educational programs to dispel myths and promote male involvement in reproductive health. Healthcare services should encourage shared responsibility and communication between partners, and professional training should address gender dynamics and the psychosocial aspects of male infertility. Future research should also investigate the long-term effects and effectiveness of psychosocial interventions across diverse settings.

## Conclusions

The findings highlight the necessity for healthcare systems to adopt comprehensive strategies that address both the medical and psychosocial aspects of male infertility. Training healthcare providers to recognize and manage the emotional challenges of male infertility, along with public education campaigns to reduce stigma, are essential steps toward improving the well-being of affected individuals.
